# Whole-body computed tomography patterns of dystrophic and metastatic soft-tissue calcification in dogs: a multicenter retrospective study

**DOI:** 10.4142/jvs.25284

**Published:** 2026-07-14

**Authors:** Hearang Lim, Sunghoon Jeon, Jinhwa Chang, Kwangyong Yoon, Byungjin Kim, Ji-Young Ban, Dongwoo Chang, Hee-Jung Jee, Namsoon Lee

**Affiliations:** 1 Section of Veterinary Medical Imaging, Veterinary Medical Center, College of Veterinary Medicine, Chungbuk National University, Cheongju 28644, Korea.; 2 Haemaru Referral Animal Hospital, Seongnam 13590, Korea.; 3 Korea Animal Medical Center, Cheongju 28651, Korea.; 4 Daejeon Animal Medical Center Soop: FOREST, Daejeon 34184, Korea.; 5 Bon Animal Medical Center, Suwon 16310, Korea.; 6 SKY Animal Medical Center, Cheonan 31110, Korea.; 7 Department of Information Statistics, Chungbuk National University, Cheongju 28644, Korea.

**Keywords:** Hypercalcemia, calcification, diagnostic imaging, retrospective studies, multicenter studies

## Abstract

**Importance:**

Differentiating between dystrophic and metastatic soft tissue calcifications in dogs is clinically essential but remains diagnostically challenging.

**Objective:**

This retrospective study aimed to categorize canine soft tissue calcifications on whole-body computed tomography (CT) based on anatomical location and pattern, determine if CT can differentiate between metastatic and dystrophic types based on these factors, and identify pattern tendencies within dystrophic subtypes.

**Methods:**

Calcifications were classified into group 1 (dystrophic) and group 2 (metastatic). Multivariable generalized estimating equation (GEE) logistic regression identified variables independently associated with metastatic calcification. Group 1 was subdivided into 1a (with mass) and 1b (without mass) to assess pattern tendencies across organs.

**Results:**

Organ location alone was not independently associated with calcification type. Instead, similar organ-specific patterns were observed across etiologic groups, indicating substantial topographic overlap; both groups 1b and 2 predominantly exhibited round/oval multifocal patterns in kidneys, and curvilinear/rim diffuse patterns in the vasculature. Conversely, group 1a showed a higher propensity for amorphous/irregular patterns within masses. In multivariable GEE analysis, focal distribution was significantly associated with metastatic calcification (adjusted odds ratio [OR], 1.18; 95% confidence interval [CI], 1.02–1.37; *p* = 0.029) compared to multifocal distribution, while curvilinear/rim shapes showed a curvilinear/rim shape was not significantly associated with metastatic calcification after adjustment (OR, 1.26; 95% CI, 0.98–1.63; *p* = 0.072) compared to round/oval shapes.

**Conclusions and Relevance:**

Whole-body CT effectively categorizes the locations and patterns of canine soft tissue calcifications and identifies distinct tendencies within dystrophic subtypes. However, due to significant organ-specific topographic overlaps, CT imaging alone is insufficient to definitively differentiate metastatic from dystrophic calcifications, necessitating comprehensive clinical and biochemical correlation.

## INTRODUCTION

Soft tissue calcification is a common yet nonspecific finding on diagnostic imaging examinations, with the etiology ranging from regional reactions against tumor to signs of systemic diseases [1]. Mineralization encompasses a broader spectrum of processes involving the deposition of minerals, including calcium, phosphate, and magnesium, in pathological and physiological contexts. Although calcification is primarily associated with pathological changes, mineralization may also occur as part of normal tissue remodeling, such as in bone formation or cartilage ossification [2,3]. For this study, the term “calcification” was used to inclusively encompass all calcium deposition processes observed in soft tissues, including those traditionally classified as mineralization, as detected on computed tomography (CT).

Calcification can occur through two distinct mechanisms, namely, dystrophic calcification and metastatic calcification. The mechanisms underlying dystrophic and metastatic calcification highlight their distinct pathological origins. Dystrophic calcification develops in tissues that have experienced damage or necrosis, even when systemic calcium levels are normal. Dystrophic calcification is commonly associated with chronic inflammation, tissue injury, or degenerative changes, as documented in multiple studies across different species [1,4]. Conversely, metastatic calcification results from high systemic calcium levels, often due to metabolic disorders like hyperparathyroidism or chronic renal disease [1,4].

Differentiating between dystrophic and metastatic calcifications is crucial to ensure diagnosis and plan effective treatment strategies in human medicine. CT images provide details regarding the location, density, and morphology of calcifications, making it invaluable for the assessment of lesion distribution and etiology of calcium deposition [1,4]. Studies have shown that these calcification patterns provide essential diagnostic insights into distinguishing between benign and malignant conditions, as well as between disease etiologies such as infections, inflammatory conditions, degenerative processes, and metabolic disorders [4,5].

However, in veterinary medicine, studies have primarily relied on radiographic imaging to evaluate calcifications, rather than CT. This reliance on radiography alone limits the detailed assessment of calcification patterns in soft tissue, reducing the diagnostic precision for understanding the distribution and characteristics of soft tissue mineralization [6]. Although recent veterinary CT studies have begun to focus on calcifications in specific organs [1,7], they remain limited in scope compared to the comprehensive, systemic evaluation seen in human medicine.

This study aimed to retrospectively analyze whole-body CT imaging data to categorize canine soft tissue calcifications according to their anatomical locations and morphological patterns. Concurrently, we sought to determine the diagnostic utility of CT in differentiating metastatic from dystrophic types based on these criteria, while characterizing distinct pattern tendencies within dystrophic subtypes (mass-associated vs. non-mass-associated). We hypothesized that whole-body CT would serve as an effective modality for comprehensive soft tissue evaluation. Specifically, we anticipated that distinct combinations of anatomical locations and calcification patterns would allow for the reliable differentiation between metastatic and dystrophic calcifications.

## METHODS

### Animals

This multicentric, retrospective study was conducted to analyze CT imaging data from client-owned dogs collected between January 2016 and September 2024. The data were obtained from five veterinary medical centers, as follows: Chungbuk National University Veterinary Teaching Hospital, Korea Animal Medical Center, Soop Daejeon Animal Medical Center, Suwon Bon Animal Medical Center, and Cheonan SKY Animal Medical Hospital. Dogs were included in the study if they had undergone whole-body CT imaging, defined as CT examinations covering the region from the head to the pelvic limbs.

Cases were included if they had undergone whole-body CT imaging for systemic evaluation and if image quality was sufficient to allow clear assessment of soft tissue calcifications. Medical records were required to include essential clinical information, including patient signalment and serum calcium measurements, to support classification of calcification type. When available, results of fine-needle aspiration, biopsy, or histopathology were reviewed to characterize mass-associated calcifications. CT examinations were excluded if CT image quality was inadequate or if critical medical data, particularly serum calcium levels, were missing or incomplete. Lesions consistent with calculi rather than soft tissue calcifications, such as luminal, gravity-dependent, well-defined, round lesions with very high attenuation values, were also excluded. We set this cutoff at ≥ 2,000 HU for the present study, with reference to attenuation values of urinary calculi reported in human CT studies [8,9]. All inclusion and exclusion decisions for the study were finalized by two diplomates of the Korean College of Veterinary Medical Imaging (DW.C and NS.L).

### Review of medical records

Comprehensive medical records were reviewed to gather clinical and diagnostic information relevant to the study. The collected data encompassed signalment (breed, sex, age, and body weight), clinical signs, and detailed hypercalcemia-related information, including serum calcium levels and concurrent diseases.

Hypercalcemia was evaluated using total serum calcium concentrations, incorporating ionized calcium measurements when available, and categorized according to established reference thresholds. Additionally, when fine-needle aspiration or biopsy was performed on mass lesions, the diagnostic results were recorded. Details regarding the underlying cause of calcifications, when available, were also noted.

### Classification of calcification groups

Serum calcium levels were analyzed to categorize dogs as hypercalcemic or normocalcemic. To minimize misclassification, hypercalcemia was classified as a total serum calcium concentration exceeding 12.0 mg/dL or when ionized calcium concentration was above the institution-specific reference range as suggested by previous studies [10,11].

Calcifications observed in normocalcemic dogs, as confirmed through serum calcium analysis, were categorized as dystrophic calcification, whereas calcifications identified in hypercalcemic dogs were classified as metastatic calcification. After excluding normocalcemic dogs with documented metabolic or endocrine diseases associated with metastatic calcification, such as chronic kidney disease (CKD) or hyperadrenocorticism, the remaining dystrophic calcifications were classified as group 1 and further subdivided into two subgroups based on the presence or absence of an associated mass lesion. Calcifications observed within or associated with growth lesions, such as neoplastic or non-neoplastic masses, including tumors, nodules, or other abnormal tissue proliferations were classified as group 1a. Conversely, calcifications located in non-mass lesions, typically arising in areas of prior tissue damage, necrosis, or inflammation were classified as group 1b. Metastatic calcification lesions occurring in hypercalcemic dogs, reflecting systemic calcium deposition were classified as group 2.

Each group included redundant occurrences, meaning that multiple calcifications within the same dog were counted to provide a comprehensive reflection of their prevalence and distribution. In particular, all calcified lesions identified in a single dog were recorded individually, even when observed in multiple anatomical sites (e.g., if calcifications occurred simultaneously in the kidney, blood vessels, and liver parenchyma of the same dog, each lesion was documented separately).

### CT examination

All CT scans, including the whole-body scans, were performed under general anesthesia using one of five different CT scanners: a 4-slice CT scanner (Hi Speed QX/I; GE Medical, USA), a 16-slice CT scanner system (Aquilion Start; Canon Medical Systems, Japan), 16-slice scanner (Alexion; Toshiba Medical Systems, Japan), 16-slice CT scanner (SOMATOM scope; Siemens, Japan), or a 64-slice CT scanner (Aquilion 64CFX; Toshiba Medical Systems). Dogs underwent CT scanning in sternal or dorsal recumbency under general anesthesia. Controlled ventilation and brief apnea following hyperventilation were used to minimize motion artifacts.

The CT images were acquired using either an axial or a helical CT unit. The slice thickness and intervals used were 0.625–2 mm with a pitch of 1.5, using the following parameters: 100–130 mAs tube, 120–140 kV tube voltage, 250–500 mm field of view, and 512 × 512 matrix. Images were reconstructed with 0.625–2 mm slice thickness.

### CT image analysis

The CT imaging data were assessed using a Digital Imaging and Communications in Medicine viewer (RadiAnt, Poland). All CT image evaluations were performed independently, in random order by one radiologist (HR.L), and organized using Microsoft Excel (Microsoft Corporation, USA). Soft tissue calcification lesions were assessed on both pre- and post-contrast images using a bone reconstruction algorithm (window width/window level, 1,500/300) and a soft tissue reconstruction algorithm (window width/window level, 400/60).

CT images were reviewed to identify and document the presence of soft tissue calcifications, focusing on the anatomical location and specific organs affected. The analysis focused on commonly affected soft tissue regions, including the urinary system, hepatobiliary system, respiratory system, gastrointestinal tract, reproductive system (female and male), cardiovascular system, other abdominal organs, and extra-parenchymal structures. For each identified calcification, the HU values were measured, and the mean HU value was calculated. Lesions with maximum HU value of ≥ 200 were recorded as calcification lesions [12,13].

Morphological characteristics were evaluated based on shape and distribution patterns (Fig. 1). The calcifications were categorized into three shape groups: round or oval, characterized by smooth, well-defined contours; amorphous, defined by irregular or fragmented shapes without clear boundaries; and curvilinear or rim-like, which appeared as arc-shaped or rim-like structure often outlining an anatomical feature. Distribution patterns were classified as focal, indicating a single localized calcified lesion; multifocal, characterized by multiple distinct calcified foci scattered within the tissue or organ; or diffuse, describing widespread calcifications uniformly distributed throughout the region of interest (ROI), defined as the area where calcification was predominantly identified.

**Fig. 1:**
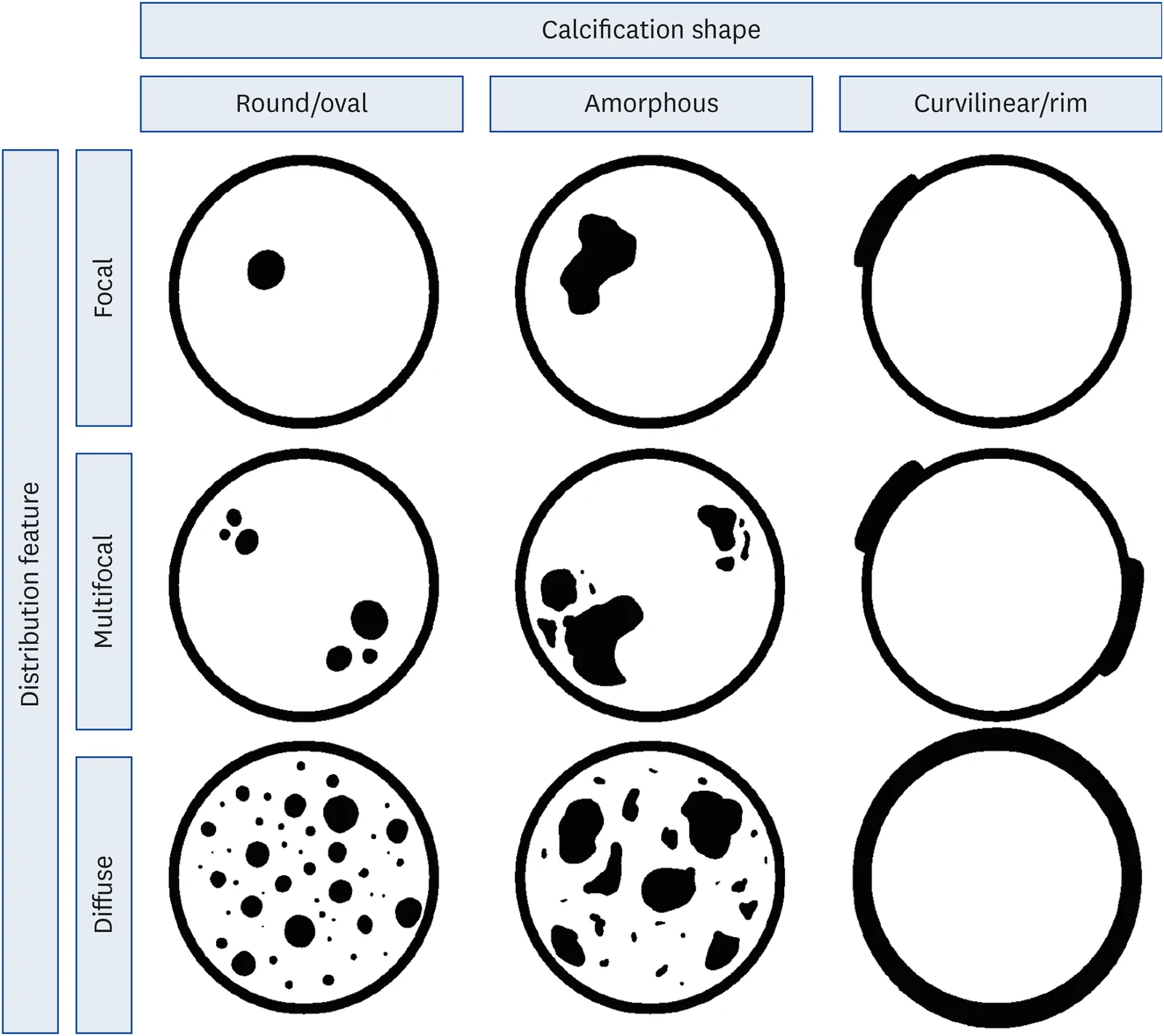
Classification matrix of soft tissue calcification patterns based on shape and distribution features. The horizontal axis represents the calcification shape, categorized into round/oval, amorphous, and curvilinear/rim patterns. The vertical axis represents the distribution feature, categorized into focal, multifocal, and diffuse patterns.

### Statistical analysis

All statistical analyses were performed using R (version 4.4.2; The R foundation, Austria). To account for the correlation among multiple calcification lesions identified within the same dog, generalized estimating equation (GEE) logistic regression models were fitted at the lesion level, with dog specified as the clustering unit. The outcome variable was calcification type, coded as dystrophic calcification (group 1) = 1 and metastatic calcification (group 2) = 0. A binomial distribution with a logit link and an independence working correlation structure were used, with robust sandwich standard errors. Observations with missing values for dog identifier, organ, calcification shape, or distribution were excluded before analysis. Organ categories with very small overall sample sizes or sparse observations in either calcification group were excluded to reduce instability in regression coefficient estimation. The organ categories retained for the GEE analysis were kidney, airway, lung, liver, vascular, mammary gland, esophagus, gastrointestinal tract, and adrenal gland. The final GEE analytic dataset consisted of 672 lesions from 461 dogs.

Unadjusted GEE logistic regression models were first fitted separately for organ, calcification shape, and distribution pattern. A multivariable GEE logistic regression model including organ, shape, and distribution was then fitted to estimate the OR for each imaging characteristic while adjusting for the other characteristics. Overall associations for each categorical variable were evaluated using Wald tests. Odds ratios (ORs) and 95% confidence intervals (CIs) were reported. Because the analyses were exploratory rather than confirmatory, no formal adjustment for multiple comparisons was applied to category-specific *p* values, and a two-sided *p* value < 0.05 was considered statistically significant.

## RESULTS

### Animals

Overall, 1,098 dogs underwent CT scans across the five veterinary medical centers included in the study. After applying the inclusion and exclusion criteria, 793 cases were selected for the final analysis. Cases were excluded due to missing patient history information (n = 135) or insufficient clinical records (n = 170). Among the 793 dogs included in the analysis, 560 were normocalcemic dogs, whereas 233 were hypercalcemic dogs. Among the normocalcemic dogs, 370 dogs initially exhibited evidence of dystrophic calcifications; however, 21 dogs with chronic renal failure or endocrine disorders associated with metastatic calcification were excluded, resulting in a final total of 347 dogs in the dystrophic calcification group, with diagnoses established using available clinicopathologic data, including blood chemistry, urinalysis, and hormone testing. A total of 448 calcification lesions were identified in group 1. These cases were further subdivided into two categories: group 1a included 93 lesions, and group 1b included 474 lesions, with multiple calcification lesions recorded per dog when present. Additionally, among the hypercalcemic dogs, 161 cases exhibited evidence of metastatic calcifications, classified as group 2, with a total of 224 metastatic calcification lesions identified (Fig. 2). Statistical analysis revealed no significant differences in age, body weight, or sex distribution among the groups. The demographic distribution is summarized in Supplementary Table 1.

**Fig. 2:**
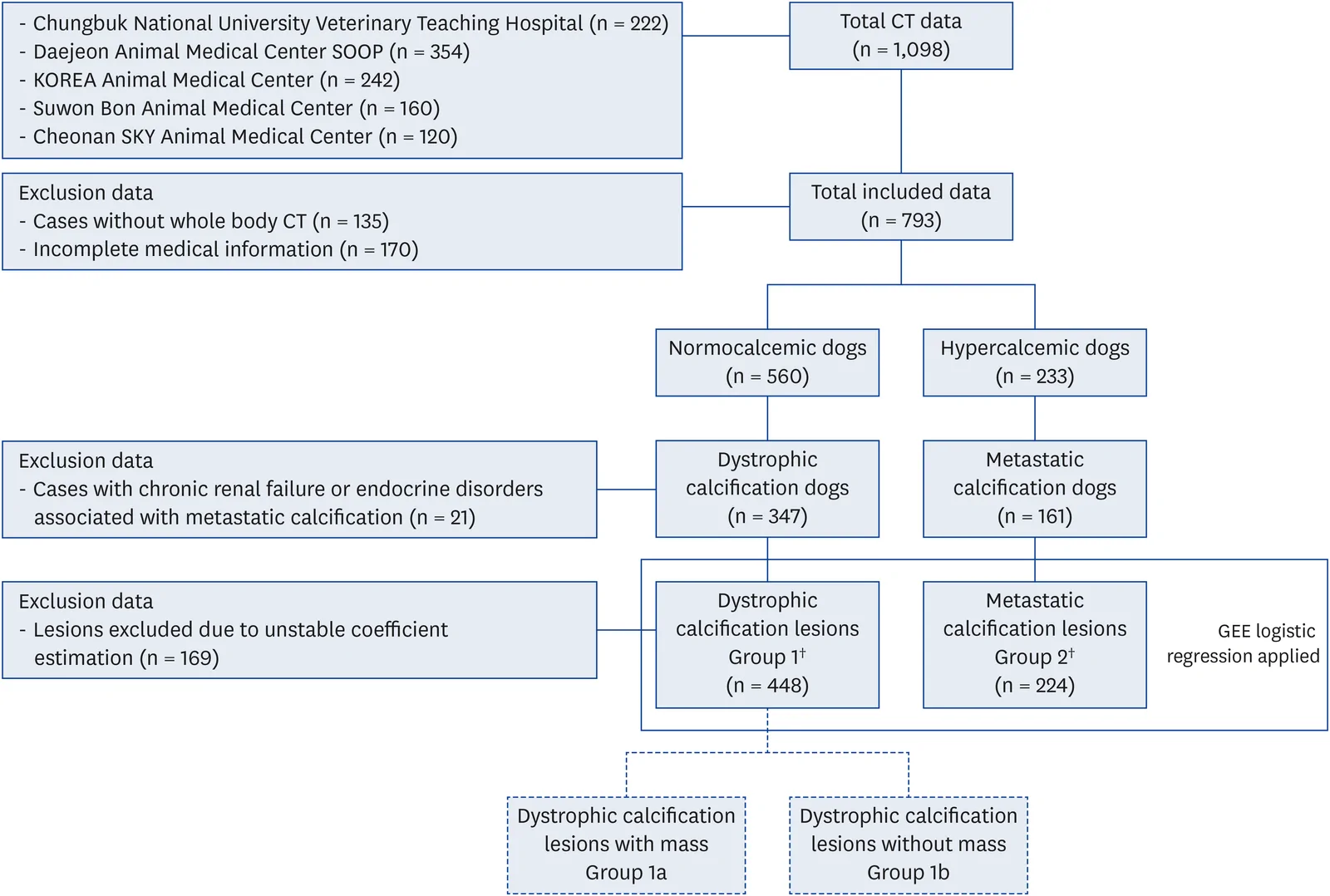
Flowchart of study inclusion criteria and classification of calcification types in dogs. Group 1 was further subdivided into group 1a (with mass lesions) and group 1b (without mass lesions); these subgroups (dashed boxes) were excluded from the main GEE analysis and instead utilized for the descriptive assessment of organ-specific calcification pattern tendencies without formal statistical testing. GEE, generalized estimating equation; CT, computed tomography. ^†^Group 1 (dystrophic calcification lesions) and group 2 (metastatic calcification lesions) were compared using a GEE logistic regression model to account for within-subject correlation among multiple lesions from the same dog.

### Anatomical distribution of calcification lesions

The anatomical distribution of soft tissue calcification lesions for groups 1a, 1b, and 2 across various organ systems is summarized in Fig. 3. Overall, the vast majority of calcifications were concentrated in the kidneys, airway, and vascular system. In the unadjusted GEE logistic regression analysis, organ location was associated with calcification type overall (*p* = 0.019). Airway lesions (OR, 0.56; 95% CI, 0.38–0.82; *p* = 0.003) and esophageal lesions (OR, 0.31; 95% CI, 0.13–0.73; *p* = 0.008) showed lower odds of dystrophic relative to metastatic calcification compared with kidney lesions. However, the overall association of organ location was no longer observed in the multivariable analysis (*p* = 0.310), and no individual organ location remained significantly associated with calcification type. The complete unadjusted GEE logistic regression results for organ locations are summarized in Table 1.

**Fig. 3:**
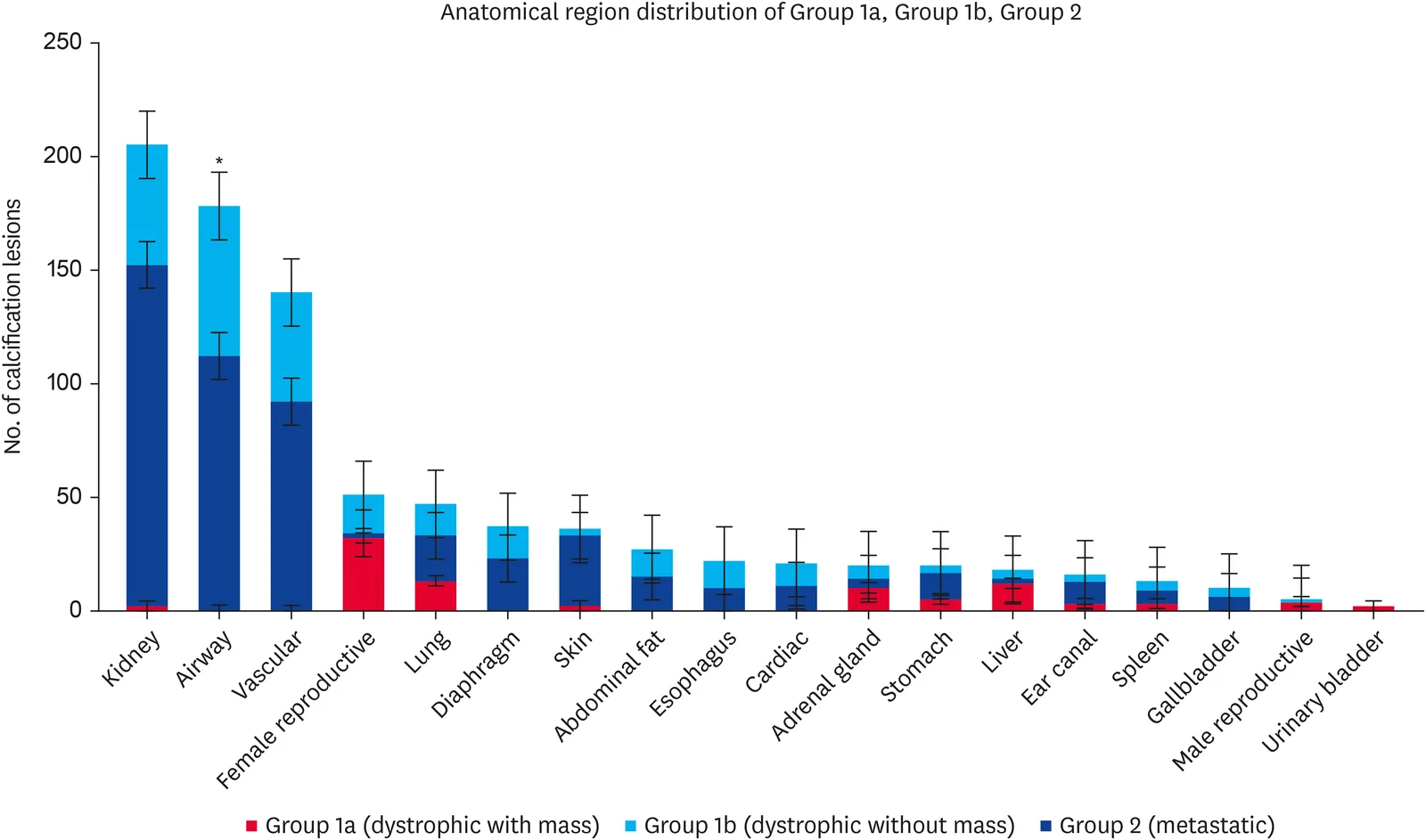
Anatomical distribution of soft tissue calcification lesions across various organ systems for groups 1a, 1b, and 2. ^*^An organ system demonstrating a statistically significant difference in group proportions in the unadjusted generalized estimating equation analysis (*p* < 0.05), which was subsequently accounted for by morphological variables in the multivariable model.

**Table 1:** Lesion-level generalized estimating equation logistic regression analyses of CT features associated with dystrophic versus metastatic calcification (groups 1 vs. groups 2)

Variable		Unadjusted OR (95% CI)	*p* value	Adjusted OR (95% CI)	*p* value
Organ			0.019		0.310
	Kidney (Ref)	1.00 (Reference)		1.00 (Reference)	
	Adrenal gland	0.88 (0.33–2.36)	0.795	0.77 (0.27–2.19)	0.617
	Airway	0.56 (0.38–0.82)	0.003	0.65 (0.34–1.24)	0.191
	Esophagus	0.31 (0.13–0.73)	0.008	0.50 (0.18–1.42)	0.194
	Stomach	2.13 (0.60–7.52)	0.240	2.16 (0.57–8.14)	0.255
	Liver parenchyma	1.05 (0.37–3.01)	0.924	0.74 (0.23–2.33)	0.602
	Lung parenchyma	0.86 (0.43–1.72)	0.667	0.70 (0.33–1.49)	0.353
	Female reproductive	0.75 (0.40–1.41)	0.375	0.50 (0.22–1.15)	0.103
	Vascular	0.69 (0.46–1.04)	0.078	0.84 (0.46–1.52)	0.566
Shape			0.007		0.056
	Round/oval (Ref)	1.00 (Reference)		1.00 (Reference)	
	Curvilinear/rim	0.63 (0.46–0.87)	0.004	0.50 (0.27–0.90)	0.021
	Amorphous	1.03 (0.60–1.79)	0.905	1.22 (0.58–2.55)	0.597
Distribution			0.047		0.003
	Multifocal (Ref)	1.00 (Reference)		1.00 (Reference)	
	Focal	0.57 (0.36–0.89)	0.014	0.55 (0.34–0.89)	0.016
	Diffuse	0.78 (0.54–1.12)	0.174	1.42 (0.86–2.33)	0.166

The outcome was calcification type, with dystrophic calcification (group 1) coded as 1 and metastatic calcification (group 2) as 0. The lesion was the unit of analysis and dog was the clustering unit. An independence working correlation structure with robust sandwich standard errors was used. ORs > 1 indicate higher odds of dystrophic relative to metastatic calcification. Reference categories were kidney for organ, round/oval for shape, and multifocal for distribution. The *p* values shown for Organ, Shape, and Distribution represent overall Wald tests. All GEE models were based on 672 lesions from 461 dogs (448 lesions from 320 dogs in group 1 and 224 lesions from 141 dogs in group 2).

### Calcification patterns

In the multivariable GEE model, distribution pattern was significantly associated with calcification type overall (*p* = 0.003). Focal distribution was associated with lower odds of dystrophic relative to metastatic calcification compared with multifocal distribution (adjusted OR, 0.55; 95% CI, 0.34–0.89; *p* = 0.016). The overall association for calcification shape did not reach statistical significance (*p* = 0.056), although curvilinear/rim-shaped calcifications showed lower odds of dystrophic calcification compared with round/oval calcifications in the category-specific comparison (adjusted OR, 0.50; 95% CI, 0.27–0.90; *p* = 0.021) (Fig. 4B). These regression results are summarized in Table 1. To further characterize calcification patterns within the dystrophic calcification cohort, group 1 was subdivided into group 1a (mass-associated) and group 1b (non-mass-associated). Topographic and morphological tendencies across the three groups (groups 1a, 1b, and 2) were descriptively evaluated for each organ system without formal statistical testing. Within the respiratory system, 46 calcification lesions were identified in the lung parenchyma. Among these, amorphous-shaped calcifications with a diffuse distribution were prominently observed in group 1a (6/13, 46%) compared to the other groups (Fig. 5A). In the cardiovascular system, calcifications were located within the cardiac and vascular walls. Of the 136 lesions recorded in the vascular walls, a curvilinear/rim-shaped morphology with a diffuse distribution was highly prevalent in both group 1b (57/88, 65%) and group 2 (29/48, 60%), indicating a strong architectural influence of the vessel wall (Fig. 5B). A total of 51 calcification lesions were identified in the female reproductive system, primarily localized to the mammary glands. In this system, amorphous-shaped multifocal calcifications were highly prevalent in group 1a (18/32, 56%) compared to the other subgroups (Fig. 5C). Lastly, 20 calcification lesions were recorded in the stomach; among these, curvilinear/rim-shaped calcifications with a diffuse distribution were predominant in group 1b (8/12, 67%) (Fig. 5D). These descriptive organ-specific patterns and distributions are comprehensively detailed in Supplementary Table 2.

**Fig. 4:**
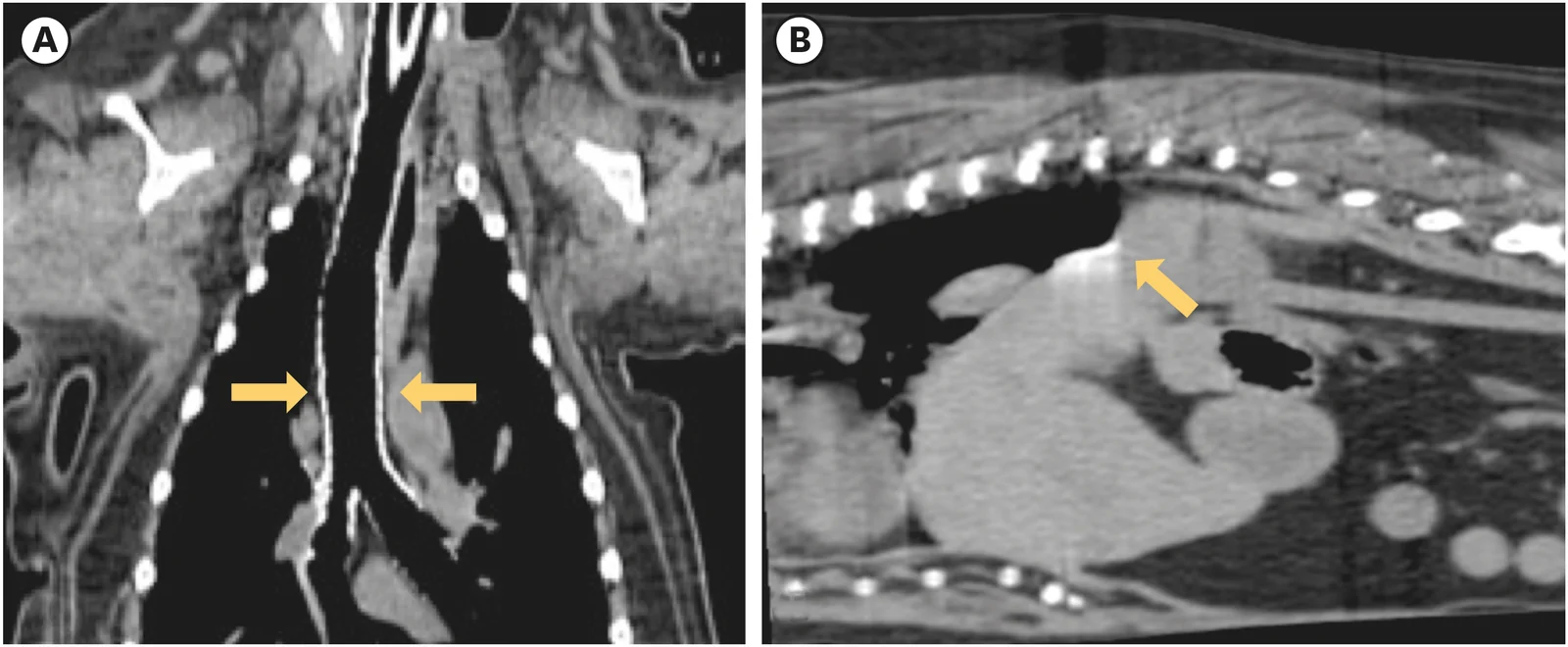
Representative CT features of curvilinear/rim-shaped calcifications in group 2. Curvilinear/rim-shaped, diffuse calcifications within the airway walls (yellow arrows) on a coronal pre-contrast CT image (A). A curvilinear/rim-shaped, focal calcification lesion within the diaphragm (yellow arrow) on a sagittal pre-contrast CT image (B). CT, computed tomography.

**Fig. 5:**
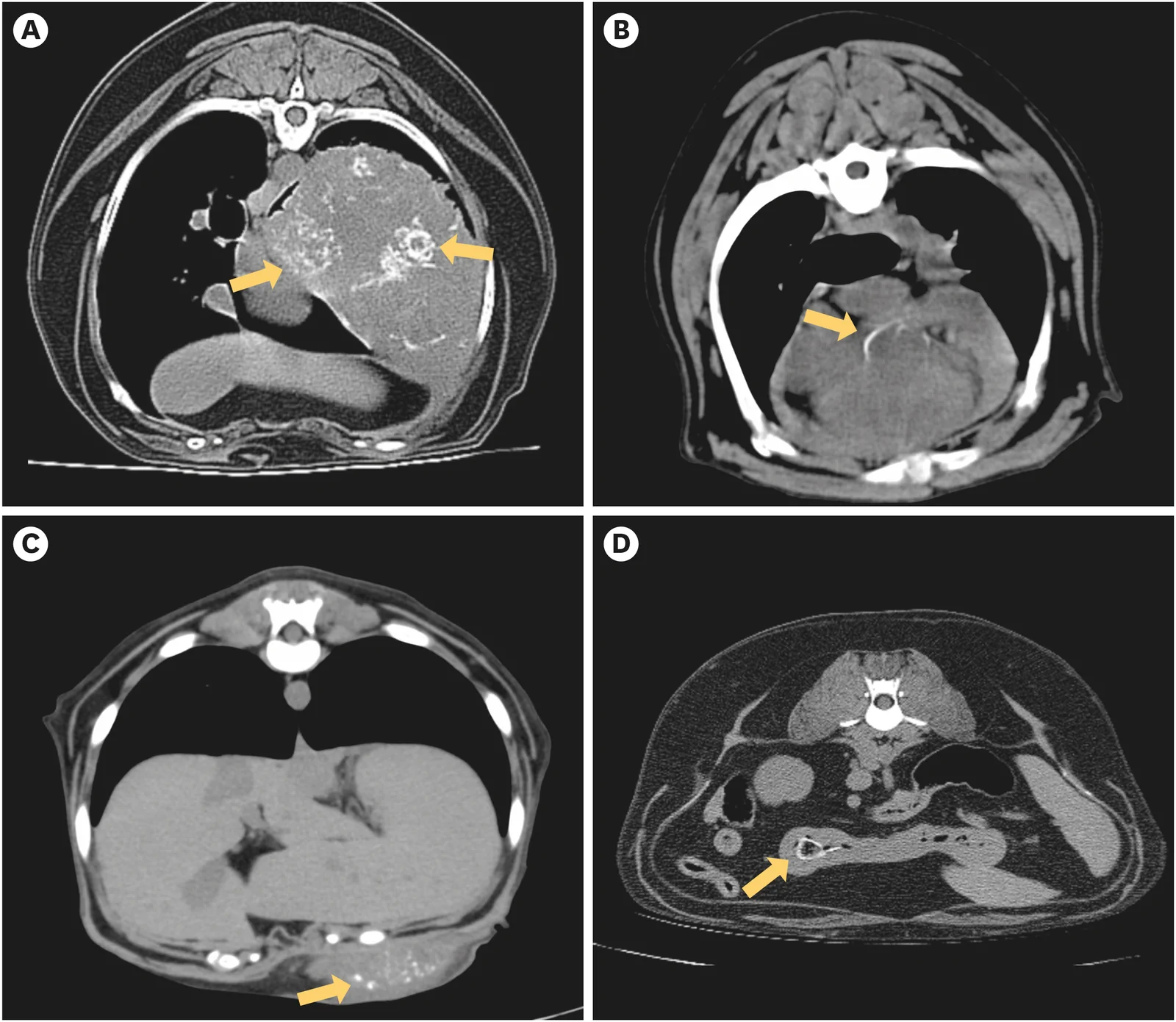
Representative CT features of soft tissue calcification patterns across different organ systems. Amorphous-shaped, diffuse calcifications within a lung parenchymal mass (yellow arrows) on a transverse pre-contrast CT image from a dog in group 1a (A). A curvilinear/rim-shaped, focal calcification lesion within the ascending aorta wall (yellow arrow) on a transverse pre-contrast CT image from a dog in group 2 (B). Round/oval-shaped, multifocal calcification lesions within a mammary gland adenocarcinoma (yellow arrow) on a transverse pre-contrast CT image from a dog in group 1a (C). A curvilinear/rim-shaped, diffuse calcification lesion within the stomach wall (yellow arrow) on a transverse pre-contrast CT image from a dog in group 1b (D). CT, computed tomography.

## DISCUSSION

In this study, whole-body CT demonstrated clear diagnostic advantages over conventional radiography by enabling a comprehensive, systemic evaluation of soft tissue calcifications. Unlike radiography, which is heavily limited by superimposition and patient size, CT allowed for the precise, concurrent characterization of anatomical location, shape, distribution, and lesion extent, providing an unprecedented whole-body map of mineral deposition in this large retrospective study.

A principal concern in analyzing lesion-level data from multi-lesion subjects is the assumption of independence, which is violated when multiple calcifications arise from the same individual. To address this, GEE logistic regression with robust sandwich standard errors and dog as the clustering unit was applied across all subjects. In the unadjusted analysis, organ location was significantly associated with calcification type overall (*p* = 0.019), with airway and esophageal involvement showing lower odds of dystrophic relative to metastatic calcification compared with kidney (airway: unadjusted OR, 0.56; 95% CI, 0.38–0.82; *p* = 0.003; esophagus: unadjusted OR, 0.31; 95% CI, 0.13–0.73; *p* = 0.008). However, the overall association of organ location disappeared in the multivariable model (*p* = 0.310). This phenomenon carries important pathophysiological and clinical implications. Under hypercalcemic states, metastatic mineral deposition in the respiratory tract is known to preferentially conform to the basement membranes of alveolar walls and bronchial structures [4,14,15]. Consequently, airway calcifications present with highly predictable curvilinear or rim-shaped contours. Our GEE model suggests that organ location alone does not independently distinguish calcification type once shape and distribution are taken into account. Clinically, this warns radiologists that identifying calcification within the airway should not trigger an automatic diagnosis of metastatic disease without considering the geometric pattern of the deposit. Among the morphological variables, distribution pattern showed a significant overall association with calcification type in the multivariable model (*p* = 0.003). In particular, focal distribution was associated with lower odds of dystrophic relative to metastatic calcification compared with multifocal distribution (adjusted OR, 0.55; 95% CI, 0.34–0.89; *p* = 0.016), indicating a greater relative association with metastatic calcification. Mechanistically, this is consistent with the pathophysiology of metastatic calcification: elevated serum calcium-phosphate products drive discrete mineral deposition at high-alkalinity sites—such as vascular smooth muscle and elastic fibers—producing focal rather than diffuse deposits [14,16]. Because our large retrospective dataset likely captured many patients during early or subclinical phases of metabolic disease, focal deposition statistically signaled a metastatic origin. Concurrently, curvilinear or rim-shaped morphology showed a category-specific association with calcification type (adjusted OR, 0.50; 95% CI, 0.27–0.90; *p* = 0.021), although the overall association for shape did not reach the nominal significance threshold (*p* = 0.056). This finding should therefore be interpreted cautiously. These contours typically form as minerals track along structural boundaries like the elastic lamina and tunica media of blood vessels or the walls of hollow visceral organs [17].

Our descriptive organ-specific analysis (Supplementary Table 2) further unmasked how local tissue architecture can overpower systemic etiology, leading to diagnostic pitfalls. For instance, within the vascular walls, a curvilinear/rim-shaped, diffuse pattern was highly prevalent in both non-mass dystrophic (group 1b: 65%) and metastatic (group 2: 60%) groups. This striking overlapping pattern suggests that the physical tubular geometry of the vasculature forces mineral tracks into identical imaging phenotypes, regardless of whether the driving force is local tissue degradation or systemic hypercalcemia. Similarly, in the kidneys, both group 1b (69%) and group 2 (49%) lesions presented predominantly as round/oval multifocal deposits, mirroring the native arrangement of renal tubules and pelvic structures. These architectural overlaps underscore the primary clinical takeaway of this study: relying solely on regional CT patterns can lead to catastrophic diagnostic errors. For example, an incidental finding of vascular or renal calcification on a staging CT cannot be reliably categorized by the radiologist based on morphology alone. Lastly, 20 calcification lesions were recorded in the stomach; among these, curvilinear/rim-shaped calcifications with a diffuse distribution were predominant in group 1b (8/12, 67%). Although metastatic calcification is typically more common in the gastric mucosa, as reported in previous studies [18], our study population demonstrated a higher prevalence of dystrophic calcification at this site, which likely reflects differences in the clinical disease spectrum or sample composition of our database.

In contrast, mass-associated dystrophic calcifications (group 1a) exhibited distinct, less structurally constrained phenotypes. In the lung parenchyma, group 1a lesions uniquely tended toward amorphous shapes with a diffuse distribution (46%), and in the female reproductive system, they predominantly presented as amorphous multifocal deposits (56%). These patterns are highly compatible with dystrophic mineralization secondary to chronic tumor remodeling, hypoxia, and dystrophic calcium precipitation within necrotic tumor cores [4]. Recognizing these amorphous, unconstrained aggregates can aid clinicians in differentiating tumor-induced necrosis from systemic metabolic calcifications.

Despite the strengths of our large dataset, several methodological limitations should be recognized. First, the retrospective design precludes establishing strict causal relationships between incidental mineralizations and underlying metabolic status. While calculi were rigorously excluded based on specific spatial and attenuation criteria (≥ 2,000 HU, luminal, and gravity-dependent positioning) to isolate true soft tissue calcifications [19], misclassification remains possible. Furthermore, metastatic calcification can occur in normocalcemic dogs with underlying conditions like hyperadrenocorticism (Cushing's syndrome) or advanced CKD due to altered calcium-phosphate products. Although we excluded normocalcemic dogs with documented CKD or endocrine histories to minimize cross-contamination, the lack of standardized, comprehensive metabolic profiles across all 1,098 cases means some metabolic lesions may have been overlooked. Lastly, longitudinal follow-up was unavailable, preventing the assessment of lesion progression or regression over time.

In conclusion, whole-body CT effectively maps canine soft tissue calcifications and establishes its diagnostic parameters. Multivariable GEE analysis demonstrated that focal distribution was significantly associated with metastatic calcification, whereas organ location showed no independent association after adjustment. Because local anatomical architecture largely dictates the imaging appearance—resulting in identical configurations of dystrophic and metastatic deposits within the kidneys and vasculature—CT features alone remain insufficient for definitive differentiation. Accurate veterinary diagnosis requires a comprehensive approach incorporating advanced CT pattern mapping, serum biochemical profiles, and clinical histories.

## SUPPLEMENTARY MATERIALS

Supplementary Table 1 Signalments and breed distribution of dogs that underwent computed tomography examination

Supplementary Table 2 Comparison of calcification patterns (shape and distribution) in the whole body computed tomography images
